# Use of a Hybrid Adeno-Associated Viral Vector Transposon System to Deliver the Insulin Gene to Diabetic NOD Mice

**DOI:** 10.3390/cells9102227

**Published:** 2020-10-02

**Authors:** Que T. La, Binhai Ren, Grant J. Logan, Sharon C. Cunningham, Neeta Khandekar, Najah T. Nassif, Bronwyn A. O’Brien, Ian E. Alexander, Ann M. Simpson

**Affiliations:** 1 School of Life Sciences, University of Technology Sydney, 15 Broadway, Ultimo, NSW 2007, Australia; Que.T.La@student.uts.edu.au (Q.T.L.); Binhai.Ren@uts.edu.au (B.R.); Najah.Nassif@uts.edu.au (N.T.N.); Bronwyn.Obrien@uts.edu.au (B.A.O.); 2 Centre for Health Technologies, University of Technology Sydney, 15 Broadway, Ultimo, NSW 2007, Australia; 3 Gene Therapy Research Unit, Children’s Medical Research Institute and Children’s Hospital at Westmead, Faculty of Medicine and Health, The University of Sydney and Sydney Children’s Hospitals Network, 214 Hawkesbury Rd, Westmead, NSW 2145, Australia; glogan@cmri.org.au (G.J.L.); scunningham@cmri.org.au (S.C.C.); nkhandekar@cmri.org.au (N.K.); ian.alexander@health.nsw.gov.au (I.E.A.); 4 Discipline of Child and Adolescent Health, Sydney Medical School, Faculty of Medicine and Health, The University of Sydney, Westmead, NSW 2145, Australia

**Keywords:** diabetes, liver, adeno-associated viral vector, transposon, gene therapy

## Abstract

Previously, we used a lentiviral vector to deliver furin-cleavable human insulin (*INS-FUR*) to the livers in several animal models of diabetes using intervallic infusion in full flow occlusion (FFO), with resultant reversal of diabetes, restoration of glucose tolerance and pancreatic transdifferentiation (PT), due to the expression of beta (β)-cell transcription factors (β-TFs). The present study aimed to determine whether we could similarly reverse diabetes in the non-obese diabetic (NOD) mouse using an adeno-associated viral vector (AAV) to deliver *INS*-*FUR* ± the β-TF *Pdx1* to the livers of diabetic mice. The traditional AAV8, which provides episomal expression, and the hybrid AAV8/*piggyBac* that results in transgene integration were used. Diabetic mice that received AAV8-*INS-FUR* became hypoglycaemic with abnormal intraperitoneal glucose tolerance tests (IPGTTs). Expression of β-TFs was not detected in the livers. Reversal of diabetes was not achieved in mice that received AAV8-*INS*-FUR and AAV8-*Pdx1* and IPGTTs were abnormal. Normoglycaemia and glucose tolerance were achieved in mice that received AAV8/*piggyBac*-*INS-FUR*/FFO. Definitive evidence of PT was not observed. This is the first in vivo study using the hybrid AAV8/*piggyBac* system to treat Type 1 diabetes (T1D). However, further development is required before the system can be used for gene therapy of T1D.

## 1. Introduction

Type 1 diabetes (T1D) is characterised by the autoimmune destruction of pancreatic beta (β) cells, resulting in a lack of insulin secretion and hyperglycaemia [1]. Currently, a patient’s blood glucose levels are controlled by multiple daily injections of insulin or by insulin pumps [2] and the development of fast and long-acting insulin analogues has provided more physiological control than older insulins [3]. However, this approach also results in susceptibility to severe hypoglycaemia, insulin resistance, and mild obesity and does not eliminate complications such as nephropathy, retinopathy, cardiovascular disorders and various neurological problems, which increase morbidity and mortality [4]. Continuous glucose sensors and insulin pumps have led to the development of the artificial pancreas, which can provide better glycaemic control [5]. However, issues such as the high costs of the systems, scar tissue associated with microneedle insertion and sensor failure limit their current usefulness [6]. Transplantation therapy of whole pancreas, human islets or combinations of islets and mesenchymal stem cells [7] are other alternatives to exogenous insulin treatment. However, the limitations of lack of donors, complications of immunosuppressive therapy and issues such as blood-mediated inflammatory reactions [8] underscore the need for alternative treatment approaches.

Gene therapy offers an alternative approach to the treatment/cure of T1D, whereby an “artificial β cell” that is capable of synthesising insulin in response to the normal metabolic signals is genetically engineered from the patient’s own cells. This approach would avoid the problem of rejection seen with both allogeneic transplantation of islets and pancreas and would release T1D patients from daily insulin injections, the risk of hypo- and hyperglycaemia episodes and the long-term chronic complications that lower quality of life and cost the community millions of dollars in patient care. As the patient’s own cells will become insulin-secreting cells, adverse allogeneic immune responses will be avoided. Additionally, there will be no requirement for the ex vivo manipulation of a patient’s liver, and subsequent adoptive transfer of artificial β cells. Hepatocytes have been shown to be suitable candidates for the generation of artificial β cells [9,10,11,12,13,14,15,16,17,18,19,20,21,22,23,24,25]. In most cases, hyperglycaemia has been ameliorated by the expression of various β-cell transcription factors in liver cells, delivered most commonly by adenoviral vectors [13,16,17,19]. However, a number of problems have been encountered using adenoviral vectors. These include the development of exocrine cells in the liver [13,16] and resultant hepatitis following the delivery of *Pdx1* [17]. Other attempts to deliver combinations of transcription factors, such as *Pdx1* and *Neurogenin 3 (Ngn3)*, improved the blood glucose levels of diabetic mice, but could not fully cure diabetes [18,19].

Our laboratory has established that the dual expression of insulin and specific β-cell transcription factors in liver cell lines and primary hepatocytes has a synergistic effect causing pancreatic transdifferentiation, storage of insulin in granules, regulated insulin secretion to glucose and other β-cell secretogogues, and, most importantly, the ability to permanently reverse diabetes [9,10,20,25]. In order to obtain consistently high transduction rates and transgene isolation to the liver in diabetic animals, we developed a novel microsurgical procedure of intervallic infusion in full flow occlusion (FFO) to deliver a lentiviral vector. This technique involved clamping the major veins and arteries to stop blood flow to the liver whilst the vector was injected into the portal circulation. This allowed the vector, containing the insulin gene, to remain isolated to the liver with resultant high transduction rates: 60% in streptozotocin (STZ)-diabetic rats [9] and 42% in spontaneously diabetic non obese diabetic (NOD) mice [10]. These results are likely attributable to the FFO technique, eliminating significant quantities of blood that would have rapidly inactivated the vector by a complement-mediated mechanism. In a world first, we have also observed the expression of human β-cell transcription factors in primary human hepatocytes engrafted into the liver of the humanised FRG mouse [25], in which chimeric human/mouse livers can be generated [26,27]. 

However, this approach of using the second-generation lentiviral vector in combination with the FFO technique has limitations due to clinical concerns, such as susceptibility to complement inactivation [28] and the invasiveness of the surgical procedure. Ultimately, a more clinically applicable vector system to deliver insulin is required for the translation of this technology. Therefore, in the present study, we endeavoured to design a protocol for the reversal of diabetes using liver-directed gene therapy without the requirement of invasive surgery. For this, we initially used a non-integrating recombinant adeno-associated vector (rAAV). These vectors are attractive candidates for gene therapy as they show long-term gene expression and lack both pathogenicity and immunogenicity.The rAAVs can transduce liver cells with high efficiency and are showing promise in clinical trials [29,30]. In this project we utilised the highly murine liver trophic type 8 capsid [31], with the incorporation of a liver specific promoter (LSP) [32] to the AAV8 vector, which allowed the systemic delivery by intraperitoneal (i.p.) injection to mouse livers. 

We transduced the livers of diabetic NOD mice via i.p. injections of AAV8-*INS-FUR* and AAV8-containing the β-cell transcription factor, *Pdx1* (AAV8-*Pdx1*), with the intention of inducing pancreatic transdifferentiation in the livers. Diabetic mice that received i.p. injections of AAV8-*INS*-*FUR* became hypoglycaemic with abnormal responses to (i.p.) glucose tolerance tests (IPGTTs). In addition, expression of β-cell transcription factors was not detected in the livers, indicating that this approach was not able to induce β-cell transdifferentiation as anticipated.

In previous successful studies, the lentiviral vector system stably incorporated the *INS-FUR* gene into liver cells [9,10,11,25]. In an attempt to investigate whether it was the episomal expression of *INS*-*FUR* using the AAV8 system that resulted in the absence of pancreatic transdifferentiation and persistence of abnormal glucose tolerance, we also employed the AAV8/*piggyBac* system, which can mediate the transposition of transgenes into the host genome [33,34,35]. As the *piggyBac* system has shown sustained gene expression in adult mice [35], we hypothesised that the AAV8-*INS-FUR*-*piggyBac* system would result in somatic integration and long-term gene expression, therby reversing their diabetes in a similar manner to the lentiviral system [9,10,25]. Expression of *INS-FUR* resulted in euglycaemia and normal IPGTTs in the mice that received AAV8/*piggyBac*-*INS-FUR* and these were subsequently subjected to FFO surgery. The *INS-FUR* gene and the pancreatic hormones, somatostatin and pancreatic polypeptide, were detected in the livers. However, wider evidence of pancreatic transdifferentiation was not seen. This is the first in vivo study using the hybrid AAV8/*piggyBac* system in an attempt to cure autoimmune T1D. However, whilst integration of the AAV8/*piggyBac* vector produced superior results compared to the episomal AAV8 system, it is apparent that the lentiviral system possesses a certain factor(s) that enables widespread pancreatic transdifferentiation to occur in the animal livers that was not seen with either AAV vector. 

## 2. Materials and Methods

### 2.1. Vector Construction and Production

#### 2.1.1. AAV Vectors

AAV vector constructs (Figure S1) were prepared using a previously reported construct [36], where a powerful liver-specific promoter drives transgene expression. New constructs were built using an In-Fusion cloning kit (Takara-bio, Scientifix Pty Ltd., Clayton, Australia), where the GFP transgene was replaced by sequences encoding *INS-FUR* or murine *Pdx1* with a downstream IRES. AAV vector stocks were produced by triple transfection of HEK 293 cells, as previously described [36]. The titre was acquired using real-time quantitative PCR (qPCR) (Table S1) [37]. The vectors were diluted with phosphate-buffered saline to the required concentration for injection. When combinations of vectors were used, the vectors were mixed and delivered in a single (i.p.) injection. 

#### 2.1.2. HIV/MSCV Lentiviral Vector 

The HIV/MSCV (HMD) lentiviral vector, which expresses the enhanced green fluorescent protein (EGFP), has a HIV/murine stem cell virus (MSCV) hybrid long-terminal repeat as the promoter [38]. The vector was produced by calcium phosphate precipitation in 293T cells using conditioned medium, as previously described [9]. The culture medium was harvested 48 h after transfection and subjected to syringe and tangential flow filtration, followed by centrifugation to pellet the vector (50,000× *g*, 2 h). Virus titre was determined by transducing 293T cells (5 × 10^5^) with serially diluted vector stocks and quantifying numbers of EGFP-positive cells by flow cytometry, as previously described [2]. Viral replication-competency was also assessed by RT-PCR [9].

### 2.2. Transduction of Liver Tissue

Female NOD mice were obtained from the Animal Resources Centre, Perth, Australia and were housed at the Ernst Facility, University of Technology Sydney, Sydney, Australia). The housing and experimental conditions complied with the Australian Code for the Care and Use of Animals for Scientific Purposes. Experiments were approved by the Animal Care and Ethics Committee, University of Technology Sydney (ETH17-1559). The mice received treatments after they had spontaneously developed diabetes (blood glucose levels ≥ 10 mmol/L for at least 3 consecutive days). 

The vector dose used for a mouse was 5 × 10^10^ vector genomes (vg). To study the effect of the AAV8-LSP system, the mice were divided into groups of seven and injected i.p. with AAV8 vectors expressing appropriate marker genes: AAV8-*INS-FUR*-mCherry or a combination of AAV8-*INS-FUR*-venus and AAV8-*Pdx1* at equivalent doses. Untreated female diabetic and non-diabetic NOD mice were used as controls. To determine whether the FFO technique had a stimulatory effect on pancreatic transdifferentiation of the livers, the surgery was performed 7 days after the mice received i.p. injections of AAV8-*INS-FUR*-venus in order to allow expression from AAV8-*INS-FUR* prior to performing the FFO technique.

To determine whether the lentiviral capsid/promoter combination was capable of stimulating pancreatic transdifferentiation in the livers expressing AAV8-*INS-FUR*-mCherry, a further group of diabetic NOD mice (n = 6) received 5 × 10^6^ transduction units (TU) of HMD/MSCV-eGFP infusion via the portal vein during FFO surgery 7 days after i.p. injections of AAV in order to enable expression from the AAV vector to develop prior to injecting the lentiviral vector.

The AAV8/*piggyBac* vector system is comprised of two different vectors: a transposon vector carrying the *INS-FUR*-mCherry construct and a transposase vector, which works by a ‘cut and paste’ mechanism [33]. To determine whether FFO surgery could induce pancreatic transdifferentiation, FFO surgery was carried out 7 days after the diabetic NOD mice received i.p. injections of the AAV8/*piggyBac-INS-FUR*-mCherry (transposon and transposase dose was 3 × 10^10^ vg and 3.5 × 10^10^ vg, respectively). It was anticipated that the mild injury induced by the FFO surgery [9,10,11,25] would stimulate hepatocyte regeneration thereby clearing the transposase to avoid the continuous excision and insertion of the transgene on the chromosomes.

### 2.3. Functional Analysis 

Mouse body weights and blood glucose levels (BGLs) were monitored daily after the AAV8 treatments. IPGTTs were performed under anaesthesia after fasting the mice for 6 h with water ad libitum. For the IPGTTs, glucose was injected i.p. at a dose of 2 g/kg body weight. Blood was collected and glucose levels were measured at 0, 5, 15, 30, 60, 90 and 120 min after i.p. glucose injection. Human insulin in sera was quantitated using an Invitron Insulin ELISA Kit (IV2-102E, Invitron Ltd., Monmouth, UK), according to the manufacturer’s protocol.

### 2.4. Microscopic Analysis

Serial frozen sections (15 µm) of the livers were prepared and fixed using acetone. Mounting medium containing DAPI (Vector Laboratories, Burlingame, CA, USA) was applied to the fixed sections to visualise nuclei. Images were acquired using a fluorescent microscope and camera (Olympus BX60, Olympus Imaging, Macquarie Park, Australia). The excitation and emission ranges of the marker genes were as follows: 400–550 and 500–650 nm for venus, and 540–590 and 550–650 nm for mCherry, respectively. 

### 2.5. Vector Copy Number Analysis 

Livers were collected for analysis of vector copy number (VCN) at the end of the experiment. Finely diced tissue pieces (25 mg) were homogenized in 500 µL of lysis buffer (10 mM Tris-Cl (pH 8), 0.1 M EDTA (pH 8), 20 mg/mL RNase A) and incubated for 1 h at 37 °C. Proteinase K (Sigma-Aldrich, North Ryde, Australia) was then added at a final concentration of 100 µg/mL, and the digestion was continued overnight at 55 °C. DNA was extracted by adding phenol/chloroform/isoamyl alcohol (25:24:1) (Thermo Fisher Scientific, Macquarie Park, Australia). The mixture was then centrifuged (16,000× *g*, 5 min), after which the aqueous phase containing the DNA was collected. This extraction process was performed twice and was followed by two extractions with chloroform/isoamyl alcohol (24:1). The DNA was precipitated with ice-cold 100% ethanol containing 1.7 M ammonium acetate and was washed with 70% ethanol. The DNA was dissolved in 10% TE buffer. The amount of DNA extracted from each of the samples was quantified using the Nanodrop spectrophotometer (Thermo Fisher, Macquarie Park, Australia).

The VCN for the mice that received AAV-*INS-FUR*-mCherry was quantified using primers and probes specific to the woodchuck hepatitis post-transcriptional regulatory element (WPRE) [39]. Quantitative PCR was carried out using Platinum^®^ *Taq* DNA polymerase enzyme (Invitrogen/ Thermo Fisher Scientific, Macquarie Park, Australia), as per the manufacturer’s instructions. WPRE primers and probe concentrations were 0.8 and 0.2 µM, respectively (Table S1). The initial denaturation was carried out at 95 °C for 10 min (1 cycle), followed by 40 cycles of 95 °C for 15 s, and 60 °C for 60 s. Fluorescence was acquired at 60 °C and all the analyses were performed on the Rotorgene 8000 system (Qiagen, Chadstone Centre, Australia). The VCN in all samples was normalised against a qPCR specific for mouse GAPDH [34]. All standards consisted of linearised plasmids. The details of the primer and probe (Sigma-Aldrich, North Ryde, Australia) sequences are presented in Table S1. 

The VCNs for the mice that received the combination of AAV-*INS-FUR*-venus and AAV8-*Pdx1* were analysed in two steps. Firstly, to determine the total VCN, the copy number of the internal ribosome re-entry site (IRES) sequence was determined using the SYBR^®^Premix Ex Taq™ system (Takara Bio, Scientifix, Clayton, Australia). Each of the samples was analysed in duplicate. The reaction (25 µL) contained 0.4 µM final concentrations of each primer, 50 ng of DNA template and 12.5 µL of the 2x SYBR premix. The reactions were carried out at 95 °C for 30 s (1 cycle), followed by 40 cycles of 95 °C for 5 s, 60 °C for 20 s, and 72 °C for 20 s. Fluorescence was acquired at 72 °C. In the next step, the copy numbers of *INS*-*FUR* and *Pdx1* were analysed separately using the respective primers. The VCN for the transposon vectors in the mouse livers was determined by real-time qPCR. The VCN was expressed as vector copies/50 ng DNA.

### 2.6. Reverse Transcriptase Polymerase Chain Reaction (RT-PCR) Analysis

For RT-PCR analysis, liver and pancreas were collected at experimental end points and frozen in dry ice. Control pancreas and liver tissues were obtained from NOD mice that did not develop diabetes. Total RNA was extracted using the MaxWell^®^RSC instrument and the MaxWell^®^RSC Simply RNA Tissue Kit (Promega, Madison, WI, USA). RNA samples were treated with DNase I (Applied BioSystems, Thermo Fisher, Macquarie Park, Australia), according to the manufacturer’s protocol. Reverse transcription was performed using the Tetro cDNA Synthesis Kit (Bioline, Everleigh, Australia) and random primers, as per the manufacturer’s protocol. PCRs were performed using GoTaq Green PCR^®^ Master Mix (Promega, Madison, WI, USA) with PCR parameters optimised for the amplification of the following genes: *Beta-Actin, INS-FUR, Pdx1, NeuroD1, Nkx2.2, Nkx6.1, MafA, Pax6, P48,* Mouse Insulin 1 and Insulin 2, *Glut 2*, pancreatic polypeptide and somatostatin (Table S2). Primers used were designed to cross intron exon boundaries to avoid amplification of any residual genomic DNA.

### 2.7. Statistical Analysis

Data were analysed using GraphPad Prism 8 software (GraphPad Software, San Diego, CA, USA). Two-way ANOVA followed by Tukey’s multiple comparison tests were performed to compare the BGLs during the IPGTTs and the weekly random BGLs of the treated mice with that of the control groups. The Mann–Whitney test was applied when comparing the vector dosages and the VCN between the experimental groups. For the mice that were transduced by a combination of *INS*-*FUR* and *Pdx1,* a paired t-test was applied when comparing the AAV8-INS-*FUR*-venus and AAV8-*Pdx1* copy numbers. The differences were considered significant when *p* < 0.05. 

## 3. Results

### 3.1. Microscopic Analysis

**Figure 1:**
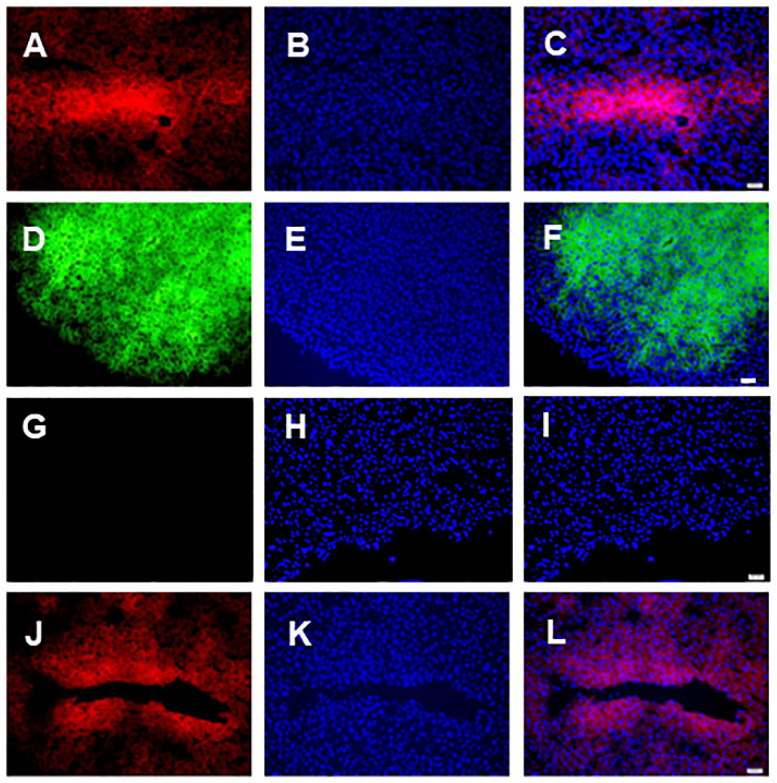
Immunofluorescence detection of marker gene expression in NOD mouse livers following transduction with AAV vectors. Frozen sections of mouse liver were prepared for immunofluorescence detection and visualised by fluorescence microscopy. (**A**) m-Cherry-*INS-FUR* immunofluorescence 9 weeks after intial transduction, (**B**) DAPI-stained nuclei of A and (**C**) is a merged image of A and B. (**D**) Venus-*INS-FUR* immunofluorescence 9 weeks after *INS-FUR* and *Pdx1* were delivered together. (**E**) shows the DAPI-stained nuclei of D and (**F**) is a merged image of D and E. (**G**) is a normal liver section showing no immunofluorescence for either venus or mCherry and (**H**) is an image of DAPI-stained nuclei of (**G**) and (**I**) is a merged image of (**G**) and (**H**). (**J**) *piggyBac*-*INS-FUR*-mCherry immunofluorescence 15 weeks after initial transduction, (**K**) is the DAPI-stained nuclei of (**J**) and (**L**) is a merged image of (**J**) and (**K**). Bar = 20 µm.

Immunoflourescent expression of the flurophores, mCherry and venus was examined in frozen sections of the transduced livers to examine transduction efficiency. Figure 1A shows expression of the mCherry marker gene (AAV8-*INS-FUR*-mCherry), Figure 1B,C show DAPI-stained nuclei and a merged image, respectively, 9 weeks after initial transduction with the non-integrating AAV8 vector. Figure 1D–F shows images of the venus marker gene (AAV8-*INS-FUR*-venus) and DAPI-stained nuclei at the experimental end point of 9 weeks after initial transduction with the non-integrating AAV8 vector. Figure 1A–F indicates widespread hepatocyte transduction as seen in our previous studies [39]. By comparison, normal liver tissue showed no expression of venus or mCherry (Figure 1G–I), with only the DAPI-stained nuclei evident. Likewise, expression of the AAV8/*piggyBac*-*INS-FUR*-mCherry transposon/transposase system was also extensive in the liver tissue (Figure 1J–L) at 15 weeks post-transduction. 

### 3.2. Delivery of AAV8 Expressing INS-FUR ± Pdx1 Fails to Reverse Diabetes

**Figure 2:**
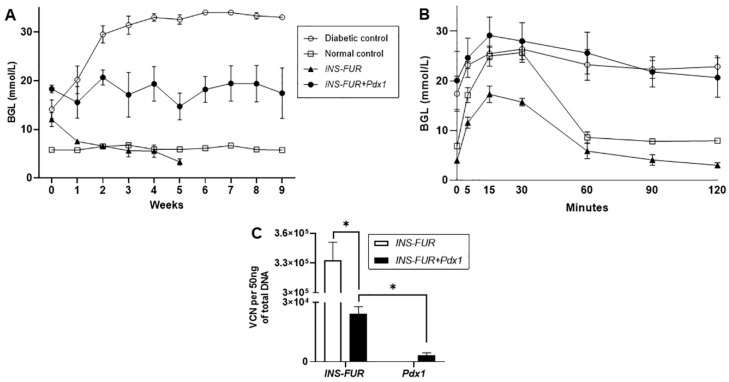
Blood glucose levels, IPGTTs and AAV8 VCNs of NOD mice after expression of *INS-FUR* alone, or with *Pdx1*, using the AAV8 vector. Transduction of NOD mice with AAV8-*INS-FUR* or AAV8-*INS-FUR* + AAV8-*Pdx1* did not reverse hyperglycaemia in diabetic mice. (**A**) The mean weekly blood glucose levels of diabetic (n = 7), normal (non-diabetic) (n = 7) and treated diabetic mice that received i.p. injections of either AAV8-*INS-FUR*-mCherry (n = 4), or AAV8-*INS-FUR*-venus + AAV8-*Pdx1* (n = 5) are shown. (**B**) Blood glucose levels following an IPGTT of diabetic (n = 3), non-diabetic (n = 7) and treated diabetic mice that received i.p. injections of either AAV8-*INS-FUR*-mCherry (n = 3) or AAV8-*INS-FUR*-venus + AAV8-*Pdx1* (n = 4). (**C**) AAV8 vector copy numbers of the diabetic mice transduced by AAV8-INSFUR-mCherry (n = 4) or AAV8-INSFUR-venus + AAV8-*Pdx1* (n = 5). Results are expressed as the means ± SEMs. * indicates a significant difference of *p* < 0.05 when comparing the VCNs.

In order to determine whether expression of *INS-FUR* alone would reverse hyperglycaemia in the diabetic NOD mice and establish normal glucose tolerance, the animals received an i.p. injection of the AAV8-*INS-FUR*-mCherry vector. The animals exhibited normalisation of BGLs on week 3, but became hypoglycaemic on week 5 (Figure 2A). The copy number of AAV8-*INS-FUR*-mCherry in the livers of these mice was 3.33 ± 0.18 × 10^5^ copies per 50 ng of DNA (Figure 2C). At all the time points during the IPGTTs, the BGLs of the mice which received AAV8-*INS-FUR*-mCherry were lower than those of the diabetic mice (*p* < 0.05) (Figure 2B). During IPGTTs, the BGLs of the mice which received AAV8-*INS-FUR*-mCherry were also lower than those of the non-diabetic control mice at 0, 5, 15, 30 and 120 min (i.e., all time points sampled excluding 60 and 90 min; *p* < 0.05) (Figure 2B). 

In an attempt to force pancreatic transdifferentiation, the β-cell transcription factor *Pdx1* [13] (AAV8-*Pdx1*) was expressed in the livers together with the *INS-FUR* gene (AAV8-*INS-FUR*-venus). Mice that received the combination of AAV8-*INS-FUR*-venus and AAV8-*Pdx1* remained hyperglycaemic (Figure 2A). At all the time points (0–120 min) during the IPGTTs, the BGLs of the mice that received the combination of *INS-FUR* and *Pdx1* were not significantly different from the diabetic mice (Figure 2B). At the end point of the experiment, the mean AAV8-*INS-FUR*-venus copy numbers in the livers were 2.41 ± 0.37 × 10^4^ copies per 50 ng of DNA, and the mean AAV8-*Pdx1* copy numbers were 3.41 ± 1.12 × 10^3^ copies per 50 ng of DNA (Figure 2C). The paired t-test showed that the AAV8-*INS-FUR*-venus copy number was significantly higher than the AAV8-*Pdx1* copy number (*p* < 0.05) in the livers of the animals. 

**Figure 3:**
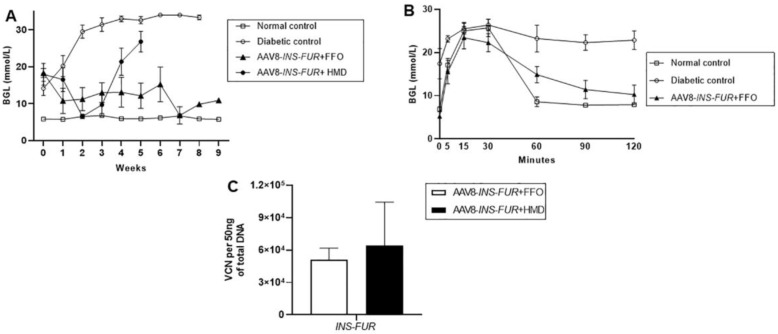
Blood glucose levels, IPGTT and AAV8 vector copy numbers of NOD mice after AAV8-mediated expression of *INS-FUR* in combination with the FFO surgical procedure, with or without dual expression of the lentiviral HMD vector. NOD mice were transduced with AAV8-*INS-FUR* in combination with the FFO surgery, as well as with dual expression of HMD. Blood glucose levels and results of subsequent IPGTT tests of the mice are shown. (**A**) Mean weekly blood glucose levels of the diabetic (n = 7), normal, non-diabetic (n = 7), and diabetic NOD mice that received i.p. injections of AAV8-*INS-FUR*-venus + FFO surgery (n = 6) and diabetic NOD mice that received AAV8-*INS-FUR*-mCherry + HMD-EGFP (n = 6). (**B**) Blood glucose levels following an IPGTT of diabetic (n = 3), non-diabetic (n = 7) and diabetic mice that received i.p. injections of AAV8-*INS-FUR*-mCherry (n = 3). The symbols for each group are indicated in the accompanying legend. Results are expressed as the means ± SEMs at each time point. (**C**) AAV8 vector copy numbers of the diabetic mice that received i.p. injections of AAV8-*INS-FUR*-venus + FFO surgey (n = 6) and diabetic NOD mice that received AAV8-*INS-FUR*-mCherry + HMD-EGFP (n = 5). Results are expressed as the means ± SEMs.

We have previously hypothesised that since liver and pancreas are from the same endodermal origin, it is likely that the FFO procedure represents an insult to the liver that stimulates differentiation of the hepatocytes to an immature phenotype. It is this differentiation process that causes expression of the β-cell transcription factors, allowing pancreatic transdifferentiation to occur in the presence of insulin and a hyperglycaemic environment [9,10,11,25]. Given the previous efficacy of using the FFO surgical technique and the lentiviral delivery of *INS-FUR* alone to reverse diabetes, we attempted to induce pancreatic transdifferentiation and normalise BGLs of diabetic mice by performing this procedure subsequent to the i.p. injection of AAV8-*INS-FUR*-venus. Unfortunately, BGLs were not normalised (Figure 3A). This group of mice had significantly lower VCNs (5.12 ± 1.06 × 10^4^ per 50 ng DNA) (Figure 3C) compared to the mice that received i.p. injections of AAV8-*INS-FUR*-mCherry (3.33 ± 0.18 × 10^5^ copies per 50 ng DNA) (*p* < 0.001) (Figure 2C). The higher BGLs and the lower AAV8 VCNs of the mice that had AAV8-*INS-FUR*-venus (i.p.) and FFO surgery, as compared to the mice that only received an i.p. injection of AAV8-*INS-FUR*-mCherry supported the hypothesis that the FFO surgery may have induced tissue damage, leading to the regeneration of hepatocytes and, therefore, the reduction in AAV8 VCNs. Despite having high BGLs, during the IPGTTs, the BGLs of the mice that received AAV8-*INS-FUR*-venus and FFO surgery were not significantly different from those of the normal controls (Figure 3B).

To determine whether the lentiviral capsid/promoter combination was capable of inducing pancreatic transdifferentiation in the livers expressing *INS-FUR*, NOD mice received HMD/MSCV-eGFP as an infusion via the portal vein during FFO surgery 7 days after having i.p. injections of AAV *INS-FUR*-mCherry. The BGLs of the mice were normalised on week 2, but they became hyperglycaemic from week 4 onwards (Figure 3A). The general health of the mice also deteriorated and symptoms of chronic hyperglycaemia, such as polyuria and polydipsia, persisted, leading to termination of the experiment before IPGGTs were performed. 

### 3.3. Reversal of Autoimmune Diabetes Using the AAV8/piggyBac-LSP-INS-FUR Vector System and FFO Surgery

**Figure 4:**
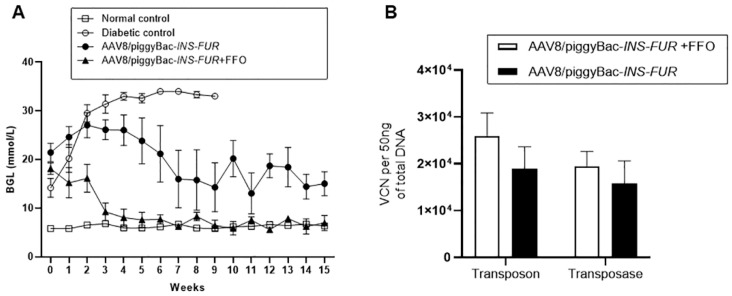
Blood glucose levels and AAV8 vector copy numbers of NOD mice after *piggyBac*/AAV8-mediated expression of *INS-FUR* and *INS-FUR* combined with the FFO surgical procedure. Diabetic NOD mice were transduced with either *INS-FUR* or *INS-FUR* in combination with the FFO surgical procedure at 7 days after transduction. (**A**) The mean weekly blood glucose levels of untreated diabetic (n = 7), normal, non-diabetic (n = 7) and treated diabetic NOD mice that received either i.p. injections of AAV8-*piggyBac/INS-FUR*-mCherry (n = 7) alone, or AAV8-*piggyBac/INS-FUR*-mCherry with FFO surgery (n = 7). (**B**) Transposon and transposase copy numbers of diabetic NOD mice that received i.p. injections of AAV8/*piggyBac*-*INS-FUR* or i.p. injection of AAV8/*piggyBac*-*INS-FUR* and FFO sugery. The results are expressed as the means ± SEMs.

The *piggyBac* transposition system, which allows for the stable expression of transgenes over time [33], was employed to determine whether the episomal (non-integrating) expression provided by the traditional AAV8 system was insufficient to stimulate pancreatic transdifferentiation in the mouse livers. Firstly, we examined if the hyperglycaemia of the diabetic NOD mice could be normalised by injection of *INS-FUR* alone. The BGLs of the mice that received an i.p. injection of the AAV8/*piggyBac*-*INS-FUR*-mCherry without FFO surgery were reduced, but normoglycaemia was not reached (Figure 4A). The transposon and transposase copy numbers in the livers of the mice that received i.p. injections of the AAV8/piggyBac-*INS-FUR*-mCherry vector system were 1.89 ± 0.05 and 1.57 ± 0.05 × 10^5^ copies per 50 ng DNA, respectively, and were not significantly different (Figure 4B).

**Figure 5:**
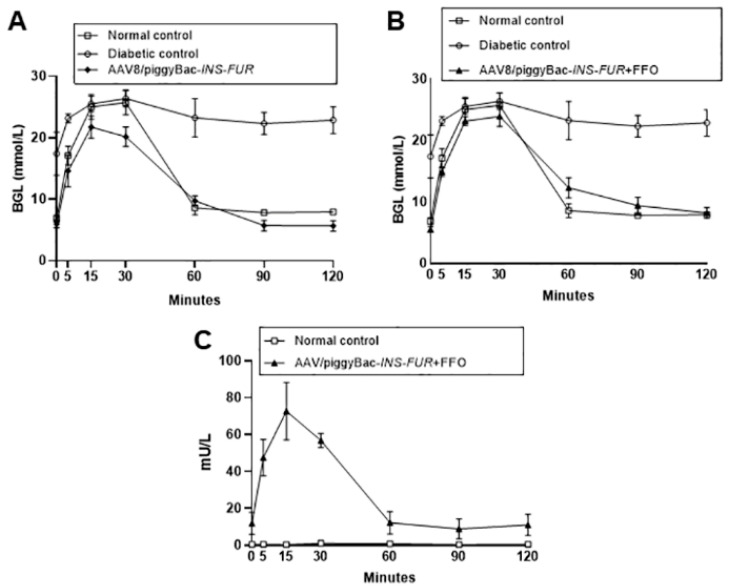
Normalisatiion of glucose tolerance of diabetic NOD mice after expression of *INS-FUR* by the integrating *piggyBac*/AAV8 vector combined with the FFO surgical procedure. Diabetic NOD mice were transduced with *INS-FUR*, or *INS-FUR* in combination with the FFO surgical procedure at 7 days after transduction, using the integrating *piggyBac*/AAV8. (**A**) Blood glucose levels following an IPGTT of diabetic (n = 3), non-diabetic (n = 7) and diabetic NOD mice that received i.p. injections of *INS-FUR* alone as AAV8-*piggyBac/INS-FUR*-mCherry (n = 7). (**B**) Blood glucose levels following an IPGTT of diabetic (n = 3), non-diabetic (n = 7) and diabetic NOD mice that received i.p. injections AAV8-*piggyBac/INS-FUR*-mCherry combined with the FFO surgery (n = 6). (**C**) Serum concentration of human insulin following IPGTT of the mice represented in (**B**), which received i.p. injections of AAV8-*piggyBac/INS-FUR*-mCherry combined with FFO surgery. Results are expressed as the means ± SEMs.

Interestingly, despite abnormal BGLs, the IPGTT results for the animals that received an i.p. injection of the AAV8/*piggyBac*-*INS-FUR*-mCherry without FFO surgery were not significantly different from those for the controls (Figure 5A). This was possibly related to this vector favouring integrated expression of *INS-FUR*. Alternatively, the constitutive expression of insulin may have reached a balanced level in response to rising glucose levels in these animals as normal IPGTTs were also seen when the non-integrating AAV8 was used to deliver *INS-FUR* (Figure 3B).

In order to determine whether the FFO procedure had a stimulatory effect on pancreatic transdifferentiation of the livers and correction of hyperglycemia, diabetic mice received the AAV8/*piggyBac*-*INS-FUR*-mCherry vector and FFO surgery 7 days later. These animals showed a reduction in BGLs at three weeks post-treatment that was then maintained at concentrations not significantly different to normal controls (experimental end point, week 15) (Figure 4A). Additionally, for animals that reverted to normoglycaemia, the BGLs during IPGTTs were not statistically different from values observed for the control mice (Figure 5B). Analysis of human insulin concentrations in sera obtained during the IPGTTs showed that the levels of human insulin for mice that had received AAV8/*piggyBac*-*INS-FUR*-mCherry and the FFO procedure peaked 15 min after glucose delivery and returned to baseline levels by 60 min (Figure 5C). These results indicated that the AAV8/*piggyBac*-*INS-FUR*-mCherry and the FFO procedure normalised BGLs for a significant period of time with normal glucose tolerance on IPGTT and human insulin peaked at levels seen in normal animals [40]. In the livers of the mice that received the AAV/*piggyBac*-*INS-FUR*-mCherry system and FFO surgery, the copy numbers of the transposon (2.6 ± 0.05 × 10^5^ copies per 50 ng DNA) and transposase (1.9 ± 0.03 × 10^5^ copies per 50 ng DNA), were not significantly different (Figure 4B).

### 3.4. RT-PCR Analysis

**Figure 6:**
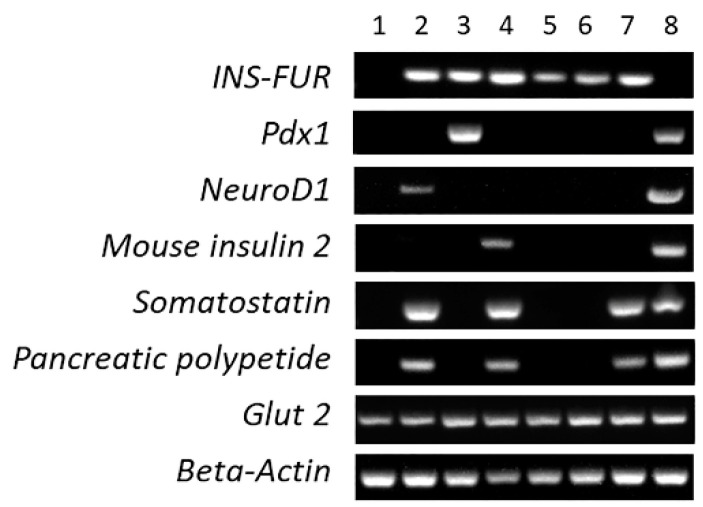
Expression of β-cell transcription factors and pancreatic hormones in the livers of transduced NOD mice. Standard end point (non-quantitative RT-PCR) analysis was conducted from RNA derived from liver tissues of the NOD mice to detect the expression of *INS-FUR*, *Pdx1, NeuroD1, mouse insulin 2, mouse somatostatin, pancreatic polypeptide, Glut 2*, and *beta-actin* (positive control) in normal mouse liver (lane 1), liver tissue from diabetic NOD mice expressing *INS-FUR* via i.p. injection of AAV8-*INS-FUR*-mCherry (lane 2), *INS-FUR* and *Pdx1* via i.p. injection of AAV8-*INS-FUR*-venus + AAV8-*Pdx1* (lane 3), AAV8-*INS-FUR*-venus + FFO surgery (lane 4), via i.p. injection of AAV8-*INS-FUR*-venus + HMD/MSCV-EGFP via the portal vein (lane 5), AAV8/*piggyBac*-*INS-FUR*-mCherry (lane 6), AAV8/*piggyBac*-*INS-FUR*-mCherry + FFO surgery (lane 7) and normal mouse pancreas (lane 8).

It can be seen from Figure 6 that expression of β-cell transcription factors was inconsistent across the experimental groups, indicative of the absence of induction of reproducible β-cell transdifferentiation in any of the treatment groups. *INS-FUR* was expressed in all samples transduced with the transgene and *Glut2* was expressed in all tissues. As expected, normal mouse pancreas expressed all genes with the exception of *INS-FUR*. The livers of mice that received AAV8-*INS-FUR*-mCherry expressed *NeuroD1*, *somatostatin* and *pancreatic polypeptide*. However, the dual transduction of *INS-FUR* and *Pdx1* resulted in expression of only *INS-FUR* and *Pdx1* and the dual transduction of AAV8-*INS-FUR*-venus + HMD/MSCV-EGFP, resulted in the expression of *INS-FUR* alone. Expression of other transcription factors indicative of β-cell transdifferentiation (*Nkx2.2, Nkx6.1, MafA, Pax6,* or *mouse insulin 1*) was not observed. The exocrine marker *p48* was also not expressed (data not shown). Similarly, no evidence of β-cell transdifferentiation was observed when the *piggy/Bac* system was used to express *INS-FUR*. In this instance, only the *INS-FUR* was expressed. Use of the *piggyBac*-*INS-FUR* system in combination with FFO surgery only resulted in expression of *somatostatin* and *pancreatic polypeptide*.

## 4. Discussion

Whilst treatment options for T1D are numerous, they are all limited in their long-term effectiveness [8] and, as a result, the search for more innovative and efficacious ways to treat/cure T1D is urgently required. Both insulin gene therapy and the reprogramming of liver cells to a β-cell phenotype have been studied by many groups as potential options [41]. The liver is considered an appropriate choice for these studies, as the liver and pancreas share a close developmental origin and the liver has great regenerative capacity. These studies have largely centred on the delivery of insulin and insulin analogues and/or β-cell transcription factors to liver cells using viral vectors, which suffer from varying multiple drawbacks. The most commonly used viral vectors are adenoviral vectors which cannot provide long-term expression of genes and are immunogenic [42]. Retroviral vectors are limited by their inability to transduce non-dividing cells and insertional mutagenesis has been problematic in a clinical trial of a severe-combined immunodeficiency patient [43]. Lentiviral vectors demonstrate long-term transgene expression but, as an integrating vector, may suffer from issues of insertional mutagenesis, although third-generation vectors have a much improved safety profile [44]. Non-integrating adeno-associated vectors show long-term expression and lack pathogenicity and immunogenicity, together with the ability to transduce liver tissues with high efficiency [31]. The AAV*piggyBac* system is known to confer stable integration, and studies with the AAV2/*piggyBac* in our laboratory have shown less frequent integrations in intragenic regions in comparison to lentiviral vectors [34] and more importantly, the integrations were not found in the loci of genes associated with hepatocellular carcinoma [36].

The β-cell transcription factor *Pdx1* has been shown to induce pancreatic transdifferentiation of liver tissue when delivered using adenoviral vectors [13,16] and some improvement in hyperglycaemia when delivered to a humanized mouse model using an AAV2 vector [22]. However, in the current study, delivery of *INS-FUR* alone (AAV8-*INS-FUR*-mCherry) or *INS-FUR* together with *Pdx1* (AAV8-*INS-FUR*-venus + AAV8-*Pdx1*), using the non-integrating AAV8 vector did not reverse hyperglycaemia and there was no evidence of expression of β-cell transcription factors that lead to pancreatic transdifferentiation. As noted in the methods, the mice received equal doses of AAV8-*INS-FUR*-mCherry, AAV8-*INS-FUR*-venus and AAV8-*Pdx1*, but the VCN of the *INS-FUR*-mCherry was significantly higher than the *INS-FUR*-venus at the conclusion of the experiments and the *INS-FUR*-venus was significantly higher than the *Pdx1.* We have much experience in quantifying the VCN by quantitative RT-PCR and are thus confident in the values presented. However, the differences in the VCN of the constructs cannot be attributed to the composition of the vectors (Figure S1) and, therefore, a definitive explanation is not possible for this observation. The 10-fold difference in the insulin vectors may be explained by the age of the mice. The NOD mice used in these experiments spontaneously developed diabetes from 12 to 26 weeks of age and it is thus not possible to isolate a diabetic cohort that is of exactly the same age. The mice used in the early experiment with the *INS-FUR*-mCherry vector that recorded VCNs 10-fold higher than those of the *INS-FUR*-venus vector averaged 16 weeks of age, whereas the second group averaged 21 weeks of age, and there is evidence that the vectors may transduce young animals more efficiently [45]. The lower transduction efficiency of the *Pdx1* vector may be due to some associated toxicity with the *Pdx1* vector, where *Pdx1*-expressing cells are lost after vector transduction. Another possible scenario may involve immune reactions against the vector. It has been recently reported that significant barriers to effective AAV2/8-insulin gene therapy in NOD mice were caused by reactivation of anti-insulin autoimmune responses as well as immune reactivity against vector components [24]. The researchers found that the efficacy of AAV-gene therapy in the NOD mouse was improved with anti-CD4 antibody treatment, indicating that T-helper subsets occurred. Future studies in NOD mice should look more closely at the immunogenicity of the vector, which may also be age dependent and consideration should be given to inducing diabetes with multiple low doses of streptozotocin (STZ) so all experimental cohorts are a similar age.

This is the first study to utilise the AAV8/*piggyBac* system to deliver human insulin to diabetic NOD mice. We showed that i.p. delivery of the AAV8/*piggyBac*-*INS-FUR* vector, significantly reduced the BGLs of spontaneously diabetic NOD mice, but did not completely reverse hyperglycaemia. By comparison, i.p. delivery of this vector, followed 7 days later by a surgical procedure that isolates the liver from the circulation (FFO), resulted in reversal of diabetes from week 3 to 15 (experimental end point), without induction of hypoglycaemia and with restoration of normal glucose toleracne. Interestingly, in both circumstances delivery of the AAV8/*piggyBac*-*INS-FUR* vector resulted in normal glucose tolerance following a 6 h fast. These results occurred without the expression of β-cell transcription factors, and, therefore, pancreatic transdifferentiation. These observations suggested that the integration of the *INS-FUR* gene alone was beneficial for the regulation of BGLs only if the FFO procedure was also used. This result was likely attributable to efficient integration of the *INS-FUR* construct (due to removal of a proportion of the transposase because of cell division) resulting in higher insulin production and reversal of hyperglcaemia. However, the integration of the *INS-FUR* gene induced by the AAV8/*piggyBac* system was insufficient to stimulate the liver-to-pancreas transdifferentiation seen with the use of the lentiviral system because the necessary pancreatic transcription factors were not also expressed. This observation suggested that the FFO surgery and the presence of a certain element(s) in the HMD vector, which were not present in the AAV8 vector, were required to induce the transdifferentiation process when *INS-FUR* was delivered.

Pancreatic transdifferentiation that results in insulin storage and regulated secretion from storage granules is one gene therapy strategy under investigation to cure T1D. It is likely that for this to occur, a “pancreatic switch” must be activated [46]. This switch may involve expression of β-cell transcription factors [9,10,11,25], transient destruction of some liver tissue by the FFO delivery technique [9,10,11,25], and/or factors present in the second generation lentiviral vector [38]. In our previous studies, the lentiviral vector likely induced pancreatic transdifferentiation in certain lineage(s) of hepatic cells that displayed plasticity, such as oval cells or stem cells, and/or took advantage of their propensity to transdifferentiate into different cell types when stressed [47]. A study by Wang et al. [19] using STZ-diabetic mice indicated that the forced liver-to-pancreas transdifferentiation was not possible utilising AAV8 vector expression of *Pdx1* and *NeuroD1*. The additional insult of an adenoviral vector that induced immune responses was required for pancreatic transdifferentiation, and some amelioration of the diabetic hyperglycaemia. Likewise, a study by Cerad-Esteban et al. [48] reported that the TALE homeoprotein, TGIF2, acts as a developmental regulator of pancreas versus liver fate in cell lines and primary rodent hepatocytes. The AAV-mediated delivery of TGIF2 first represses hepatic identity and initiates a ‘switch’ that turns on a pancreatic cell identity. We saw a similar pattern in our earlier study in NOD mice using the lentiviral vector to deliver *INS-FUR*, where there was significant upregulation of key β-cell transcription factors (*Pdx1, NeuroD1* and *Neurog3*), and significant down regulation of hepatic markers (*C/EBP-β, G6 PC, AAT* and *GLUI*) at 7 and 10 days post-transduction of the livers, which was maintained until the experimental end point (150 days) [10]. 

Based on our work, it would appear that for AAV vectors to induce liver-to-pancreas transdifferentiation an additional factor(s), such as concomitant immune responses, a minor insult, or a developmental regulator, is required. A combination of the AAV8/*piggyBac* system and a cocktail of β-cell transcription factors may warrant future investigation [49]. The current study suggests that, with further development of the AAV vector system and a better understanding of the pancreatic transdifferentiation process, the integrating AAV8/p*iggyBac* system may be useful to at least satisfy basal insulin requirements, and pancreatic transdifferentiation may not be required to achieve some advantageous clinical outcomes. Such outcomes may also be achieved with the use of inducible promoter systems such as the Tet-off system that has been shown to regulate insulin delivered by an AAV8 system in diabetic NOD.cg-Prkdcscidll2 rgtm1 Wjl/szJ mice [23]. Non-viral delivery mechanisms such as insulin constructs in minicircle DNA [21] which resulted in glucose-regulated insulin production from rat livers is a promising system that avoids possible complications of viral vectors. Haematopoetic stem cell-mediated gene therapy can produce a tolerogenic environment for islets and prevent destruction on transplantation, by halting antigen-specific memory T-cell responses [50]. This is one of many other possible gene therapy technologies being examined to treat/ cure T1D.

## Supplementary Materials

The following are available online at https://www.mdpi.com/2073-4409/9/10/2227/s1, Figure S1: AAV vector maps; Table S1: Primer and probe sequences used for the quantitation of vector and transcript copy number; Table S2: Primer sequences for the detection of target transcripts by RT-PCR.
