# Supplementary material for: Use of a Hybrid Adeno-Associated Viral Vector Transposon System to Deliver the Insulin Gene to Diabetic NOD Mice
Source: Cells. 2020 Oct 2;9(10):2227. doi: 10.3390/cells9102227 (PMC7600325; doi:10.3390/cells9102227)
Supplement: Supplementary file 1 [file cells-09-02227-s001.zip › Supplementary materials La et al 01.10.20/Figure S1 new.pdf]

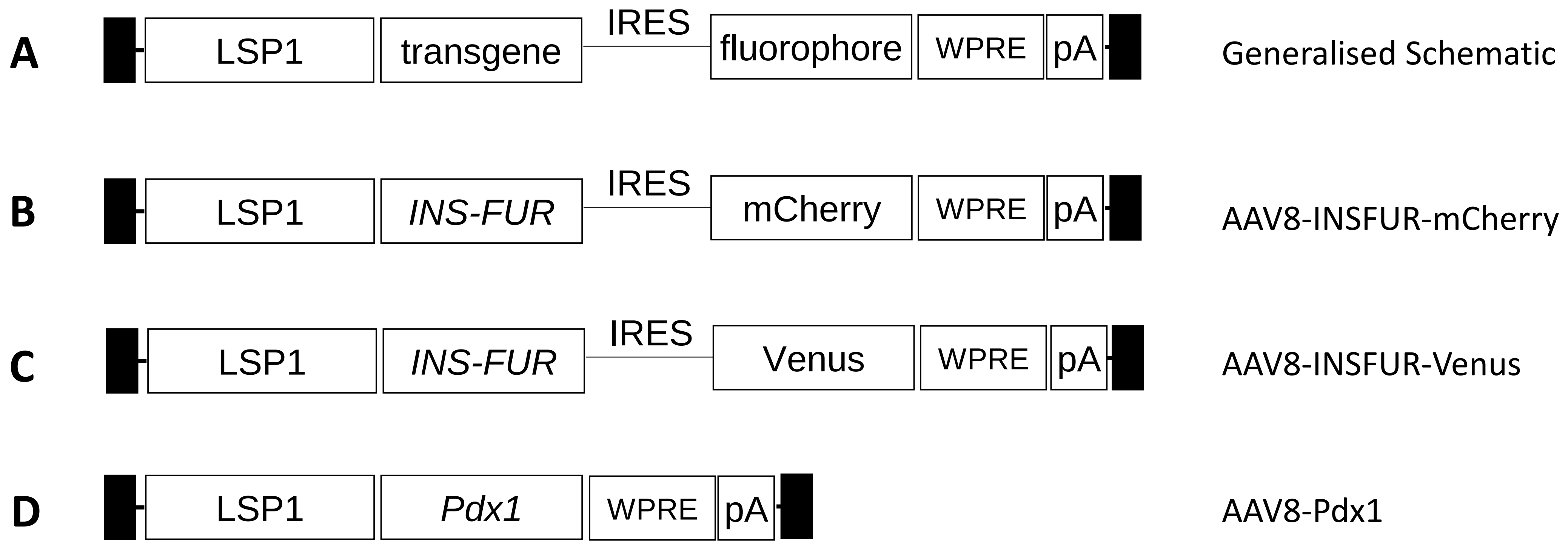

Figure S1 Schematic representation of the AAV8 construct maps used. In each construct, the transgene was under the control of the liver specific promoter. **A.** Generalised schematic map, **B.** AAV8-*INS-FUR*-mCherry, **C.** AAV8-*INS-FUR*-Venus and **D.** AAV8-*Pdx1*. **Key:** Black filled squares represent the ITR sequences; LSP1 represents the liver specific promoter; the transgenes used were either human INS-FUR or murine *Pdx1*; IRES represents the internal ribosome entry site; the fluorophores used were either mCherry (red) or Venus (green); WPRE represents a Woodchuck Hepatitis Virus (WHP) Posttranscriptional Regulatory Element; pA represents a polyadenylation signal.
