# Supplementary material for: Use of a Hybrid Adeno-Associated Viral Vector Transposon System to Deliver the Insulin Gene to Diabetic NOD Mice
Source: Cells. 2020 Oct 2;9(10):2227. doi: 10.3390/cells9102227 (PMC7600325; doi:10.3390/cells9102227)
Supplement: Supplementary file 1 [file cells-09-02227-s001.zip › Supplementary materials La et al 01.10.20/Table S1.pdf]

Table S1

Primer and probe sequences used for the quantitation of vector and transcript copy number

Primers	Primers	Sequences 5'-3'
GAPDH	Forward	ACGGCAAATTCAACGGCAC
	Reverse	TAGTGGGGTCTCGCTCCTGG
IRES	Forward	CTAACGTTACTGGCCGAAGC
	Reverse	AGGAACTGCTTCCTTCACGA
Transposase	Forward	TGGGAAGAGGAACACAGACC
	Reverse	TTGTTTGATCGCACGGTTCC
INS-FUR	Forward	CAAGAGAGAGGCCGAGGAC
	Reverse	AGCTGGTACAGGCTGCAGAT
PDX1	Forward	GAACCCGAGGAAAACAAGAG
	Reverse	GACGGTTTTGGAACCAGAT
WPRE	Forward	CCGTTGTCAGGCAACGTG
	Reverse	AGCTGACAGGTGGTGGCAAT
	Probe	5'-FAM-TGCTGACGCAACCCCCACTGGT-BHQ1-3'
