# Supplementary material for: Use of a Hybrid Adeno-Associated Viral Vector Transposon System to Deliver the Insulin Gene to Diabetic NOD Mice
Source: Cells. 2020 Oct 2;9(10):2227. doi: 10.3390/cells9102227 (PMC7600325; doi:10.3390/cells9102227)
Supplement: Supplementary file 1 [file cells-09-02227-s001.zip › Supplementary materials La et al 01.10.20/Table S2.pdf]

**Table S2**

**Primer sequences for the detection of target transcripts by RT-PCR**

| Genes | Primers | Sequences 5’-3’ |
|---|---|---|
| Beta-actin | Forward | ATGGAGGGGAATACAGCCC |
| | Reverse | TTCTTTGCAGCTCCTTCGTT |
| INS-FUR | Forward | CGCTTTCGTGAACCAGCAC |
| | Reverse | AGTTCCACCTGTCCGACCT |
| *Pdx1* | Forward | CCAGTGGGCAGGAGGTGCTTACA |
| | Reverse | CTCTGCATTCATGGCTTCAA |
| *NeuroD1* | Forward | GCTCCAGGGTTATGAGATCG |
| | Reverse | CTCTGCATTCATGGCTTCAA |
| Nkx.2.2 | Forward | ACTTGGAGCTCGAGTCTTGG |
| | Reverse | AGCCCTTTCTACGACAGCAG |
| *Nkx6.1* | Forward | CCGAGTCCTGCTTCTTCTTG |
| | Reverse | TATTCTCTGGGGATGACGGA |
| *MafA* | Forward | CTCTGGAGCTGGCACTTCTC |
| | Reverse | AGGAGGTCATCCGACTGAAA |
| *Pax6* | Forward | CTTCATCCGAGTCTTCTCCG |
| | Reverse | AGTTGGTGTTCTCTCCCCCT |
| *P48* | Forward | GAAGGTTATCATCTGCCATCG |
| | Reverse | GGGTGGTTCGTTCTCTATGTT |
| Mouse insulin 1 | Forward | TGGCTTCTTCTACACACCCAAG |
| | Reverse | ACAATGCCACGCTTCTGC |
| Mouse Insulin 2 | Forward | GAGTCCCACCCCACCCAG |
| | Reverse | TCCACTTCACGGCGGGACA |
| Pancreatic polypeptide | Forward | GCATAGTCGCCTGGGTACAT |
| | Reverse | CCAACACTCACTAGCTCAGCA |
| Somatostatin | Forward | GCTGCGCTCTGCATCGTCCT |
| | Reverse | GGCCAGTTCCTGTTTCCCGGT |
| Glut 2 | Forward | TCTTCACGGCTGTCTCTGTGCT |
| | Reverse | AGCAGCACAAGTCCCACCGAC |
